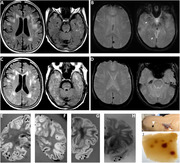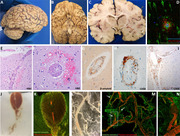# Fatal Iatrogenic Cerebral Amyloid‐Related Encephalitis in a woman treated with lecanemab for Alzheimer’s disease

**DOI:** 10.1002/alz.090928

**Published:** 2025-01-09

**Authors:** Elena Solopova

**Affiliations:** ^1^ Vanderbilt University Medical Center, Nashville, TN USA

## Abstract

**Background:**

We report the case of a 79‐year‐old woman with Alzheimer’s disease who enrolled in a clinical study of lecanemab. After the third, biweekly infusion she suffered a seizure followed by aphasia and progressive encephalopathy. Magnetic resonance imaging revealed multifocal cerebral edema and an increased burden of cerebral microhemorrhages compared to pre‐trial imaging. She was treated with an antiepileptic regimen and high‐dose intravenous corticosteroids but continued to worsen and died after five days.

**Methods:**

Post‐mortem paraformaldehyde‐fixed coronal brain slices were embedded in degassed agarose solution and scanned with human clinical MRI scanners at 3 and 7 Tesla field strength. Free‐floating sections were immunostained using validated primary and corresponding secondary antibodies and imaged using a confocal laser‐scanning microscope. For three‐dimensional lightsheet microscopy, tissue blocks were embedded in CLARITY acrylamide hydrogel, optically cleared and stained with fluorescent probes and antibodies.

**Results:**

Post‐mortem MRI confirmed extensive microhemorrhages in the temporal, parietal and occipital lobes. The autopsy confirmed the presence of two copies of APOE4, a gene associated with a higher risk of Alzheimer’s disease, and neuropathological features of moderate severity Alzheimer’s disease and severe cerebral amyloid angiopathy with perivascular lymphocytic infiltrates, reactive macrophages and fibrinoid degeneration of vessel walls. There were deposits of β‐amyloid in meningeal vessels and penetrating arterioles with numerous microaneurysms.

**Conclusion:**

We conclude that the patient likely died as a result of severe cerebral amyloid‐related meningoencephalitis.